# Soy protein supplementation does not cause lymphocytopenia in postmenopausal women

**DOI:** 10.1186/1475-2891-5-12

**Published:** 2006-04-11

**Authors:** Do Y Soung, Anagha Patade, Dania A Khalil, Edralin A Lucas, Latha Devareddy, Kathryn A Greaves, Bahram H Arjmandi

**Affiliations:** 1 Department of Nutrition, Food and Exercise Sciences, Florida State University, Tallahassee, FL, USA; 2 Department of Nutritional Sciences, Oklahoma State University, Stillwater, OK, USA; 3 The Solae Company, St. Louis, MO, USA

## Abstract

**Background:**

The health benefits of soy isoflavones have been widely investigated; however, there are some concerns as to whether soy isoflavones, similar to ipriflavone, a synthetic isoflavone, cause lymphocytopenia in postmenopausal women. Hence, the purpose of this study was to investigate the extent to which 12-month supplementation of 25 g soy protein containing 60 mg isoflavones alters lymphocyte counts or other hematological parameters in postmenopausal women who were not on hormone replacement therapy.

**Methods:**

Eighty-seven postmenopausal women were randomly assigned to receive either soy protein or an equivalent amount of control protein devoid of isoflavones. Fasting venous blood was collected at baseline and at the end of twelve month study period for complete blood count analyses.

**Results:**

Between the two treatment groups, the percent changes in hematological parameters, including lymphocytes, were not different. While women consuming the soy supplement had an increase in mean corpuscular hemoglobin concentration (MCHC) and red cell distribution width index (RDW; a marker of reticulocytes), women consuming the control diet had higher percentage of only MCHC.

**Conclusion:**

Overall, the results of the present study indicate that consumption of 25 g soy protein containing 60 mg isoflavones daily for one year does not cause lymphocytopenia.

## Background

Soy protein has been reported to have positive effects on cardiovascular [1-3] and skeletal health [4-7] in postmenopausal women. These health benefits of soy can be, in part, attributed to its isoflavone content. For example, genistein, the major isoflavone in soy, has been shown to improve cardiovascular health by modulating lipid profile and improving endothelial function in postmenopausal women [1-3]. In terms of effects on bone, one-year supplementation of the isolated isoflavone, genistein, has been reported to significantly enhance bone mineral density of femur and lumbar spine in early postmenopausal women in comparison to the placebo groups [7]. These favorable effects of genistein on skeletal health have been credited to its bone forming ability [7] while at the same time inhibiting bone resorption [6,7]. However, to date, the effects of soy and its isoflavones in preventing bone loss in postmenopausal women remain inconclusive. The findings of animal and clinical findings have ranged from no significant changes [4,5,8-12] to a slight increase [4,13-15] in BMD.

Despite the reported health benefits of soy isoflavones, there are some safety concerns about their long-term use [16,17]. These arose as a consequence of findings from a multicenter study [16] which indicated that 13.2% of 234 postmenopausal women who received 600 mg ipriflavone, a synthetic isoflavone, daily for three years developed subclinical lymphocytopenia (<0.5 × 10^3^/mm^3^). Similarly, Agnusdei and Bufalino [17] observed that approximately 3% of their subjects had lymphocyte numbers that fell outside the normal range after taking ipriflavone for two years. Ipriflavone, 7-isopropoxy-3-phenyl-4H-1-benzopyran-4-one, is a synthetic isoflavone whose metabolites include daidzein, a soy isoflavones; hence it is plausible that soy isoflavones may also have negative effects on hematological parameters including lymphocyte numbers. In contrast, findings from our rat study [18] indicated that isoflavones restored the ovariectomy-induced increase in lymphocytes and other hematological parameters to normal levels. Therefore, the purpose of this study was to examine the effects of a one-year supplementation of 25 g soy protein containing 60 mg isoflavones on hematological parameters including lymphocytes in postmenopausal women.

## Methods

### Study participants and design

This study was part of a one-year clinical trial examining whether 25 g soy protein supplementation had beneficial effects on bone mineral content and density of postmenopausal women [19]. In this part of the study, we report the effects of 25 g soy protein supplementation on hematological parameters including lymphocytes. Details about the study participants, design, anthropometric measurements, and dietary assessments were previously described [19]. The protocol was approved by the Institutional Review Board at Oklahoma State University. Briefly, postmenopausal women 65 years of age or younger, not on hormone replacement therapy (HRT), were recruited for this study. Women with any bone-related prescription medications or herbal supplements (i.e., soy isoflavones) and with disorders such as cancer, hypo- or hyperthyroidism, liver- or gastrointestinal- diseases, insulin-dependent diabetes mellitus, pelvic inflammatory disease, and endometrial polyps were excluded.

A complete medical history was obtained at the beginning of the study for each subject and one-week food frequency questionnaires and physical activity were completed via interview by a registered dietitian at the beginning and at the end of the treatment period. Eighty seven qualified volunteers were randomly assigned to receive 25 g of either control or soy protein daily for a period of one year. The soy protein foods provided 60 mg of isoflavones daily in the form of snack bars, drink mixes, and cereal (DrSoy Nutrition; Irvine, CA). The control foods were devoid of soy isoflavones and consisted of a peanut protein-based snack bar, a milk protein-based drink mix, and a wheat protein-based cereal. In order to monitor compliance, subjects were asked to return any unused supplement and record their intake on a study calendar.

### Blood collection and assessment of hematological parameters

Overnight fasting venous blood was collected in vacutainer tubes with EDTA as an anti-coagulant (Fisher Scientific) at the beginning and end of the study. Samples were analyzed for white blood cells (WBC), percentage and actual number of differential WBC, i.e., lymphocytes (LYM), monocytes (MON), neutrophils (NEU), eosinophils (EOS), basophils (BAS), red blood cells (RBC), hemoglobin (Hb), hematocrit (Hct), mean corpuscular volume (MCV), mean corpuscular hemoglobin (MCH), mean corpuscular hemoglobin concentration (MCHC), red cell distribution width index (RDW), platelet count (PLT), mean platelet volume (MPV), plateletcrit (PCT) and platelet distribution width (PDW) by an automated combined impedance-light focusing hematology counter (Pentra 120 Retic Hematology Instrument, ABX Diagnostics, Irvine, CA). The intra- and inter- assay coefficient of variations (CV) are 5.2 and 6.4%, 1.8 and 2.9%, 1.8 and 2.8%, 2.1 and 2.9%, and 6.6 and 7.8% for WBC, RBC, Hb, Hct, and PLT, respectiviely.

### Statistical analyses

Analysis of variance method was used to analyze the data with PROC GLM in PC SAS (Version 9.1, SAS Institute, Cary, NC). Fisher's Protected Least Significant Difference Procedure was used to determine which means were significantly different (*P *< 0.05). Data are reported as least square mean ± standard error (SE).

## Results

Subject characteristics are presented in Table 1. Of the 87 women recruited, only 62 completed the study. The number of subjects that dropped out of the study was similar between the two groups, 31% and 27% for the control and soy group, respectively (Table 2).

**Table 1:** Subject characteristics at baseline and after one year of supplementation with soy and control foods

Measures	Control (n = 27)			Soy (n = 35)		
	Baseline	Final	Change (%)	Baseline	Final	Change (%)
Age (yrs)	56.1 ± 1.1			53.3 ± 0.9		
Years since menopause	6.43 ± 1.09			5.00 ± 0.98		
BMI (kg/m^2^)	27.3 ± 1.0	28.2 ± 1.0	+ 3.33	28.6 ± 0.9	29.0 ± 0.9	+ 1.61

Data represent least square mean ± SE.

BMI = body mass index.

**Table 2:** Reasons for dropping out of the study

Reason	Number of Subjects	
	Control	Soy
Medical conditions preventing continuation in the study	1	2
Starting hormone replacement therapy	3	1
Non compliance with study protocol		2
Dislike of the volume or flavor of the food	3	
Gastrointestinal side effects	2	
Food was causing headaches	1	
Personal reasons	2	3
No particular reason for dropping out of study		5

Total dropouts	12	13

Data represent least square mean ± SE. BMI = body mass index.

In order to assess treatment effects, comparisons were made between final and baseline values for hematological parameters in each treatment group. There were no significant differences between the control and soy groups in any of the hematological parameters when values were expressed as percent change from baseline (Table 3, 4, 5). However, there were differences in some of the hematological parameters when comparing baseline versus final values. Both soy and control supplements significantly increased the absolute number and distribution of basophils (Table 3). Although the increase in absolute number and distribution of basophiles were significant, it is hard to interpret these findings as automated basophile counts have high CVs. Only soy supplementation somewhat (*P *< 0.087) lowered the percentage of monocytes (Table 3). Soy supplementation was also able to significantly increase MCHC and RDW, but women who received the control regimen had only higher MCHC (Table 4). No changes were observed in any of the platelet parameters as a result of dietary treatment (Table 5).

**Table 3:** Effect of one year supplementation of soy or control foods on total and the distribution of white blood cells

Measures	Control (n = 27)			Soy (n = 35)		
	Baseline	Final	Change (%)	Baseline	Final	Change (%)
WBC (×10^3^/mm^3^)	5.74 ± 0.32	5.78 ± 0.32	+ 1.47	5.54 ± 0.28	5.74 ± 0.29	+ 4.05
LYM (×10^3^/mm^3^)	1.83 ± 0.09	1.79 ± 0.09	+0.47	1.81 ± 0.08	1.80 ± 0.08	+0.99
LYM (%)	32.5 ± 1.6	32.3 ± 1.6	-0.47	33.8 ± 1.4	33.1 ± 1.4	-0.62
MON (×10^3^/mm^3^)	0.489 ± 0.035	0.493 ± 0.036	+5.39	0.535 ± 0.031	0.496 ± 0.032	-2.81
MON (%)	8.61 ± 0.46	8.68 ± 0.48	+3.80	9.76 ± 0.41	8.63 ± 0.42	-6.19
NEU (×10^3^/mm^3^)	3.12 ± 0.25	3.15 ± 0.26	+0.96	2.91 ± 0.23	3.10 ± 0.23	+9.55
NEU (%)	53.7 ± 1.8	52.8 ± 1.8	-1.43	51.3 ± 1.6	52.2 ± 1.6	+3.54
EOS (×10^3^/mm^3^)	0.176 ± 0.022	0.190 ± 0.022	+14.4	0.186 ± 0.020	0.174 ± 0.020	+4.11
EOS (%)	3.12 ± 0.33	3.34 ± 0.34	+11.3	3.31 ± 0.29	3.07 ± 0.30	+0.97
BAS (×10^3^/mm^3^)	0.118 ± 0.012	0.157 ± 0.013^†^	+53.8	0.101 ± 0.011	0.158 ± 0.011^†^	+103
BAS (%)	2.11 ± 0.25	2.96 ± 0.26^†^	+62.7	1.89 ± 0.22	2.95 ± 0.23^†^	+95.6

Data represent least square mean ± SE.

† signifies that final values were significantly different (*P *< 0.05) from their baseline values. No differences were observed between the soy and control groups when parameters where expressed as change (%) from baseline.

WBC = white blood cells; LYM = lymphocytes; MON = monocytes; NEU = neutrophils; EOS = eosinophils; BAS = basophils.

**Table 4:** Effect of one year supplementation of soy or control foods on red blood cells and related parameters

Measures	Control (n = 27)			Soy (n = 35)		
	Baseline	Final	Change (%)	Baseline	Final	Change (%)
RBC (×10^6^/mm^3^)	4.73 ± 0.07	4.73 ± 0.08	-0.20	4.51 ± 0.07*	4.55 ± 0.07	+ 0.98
Hb (g/dl)	13.6 ± 0.2	13.6 ± 0.2	+0.09	13.2 ± 0.2	13.5 ± 0.2	+2.91
Hct (%)	40.4 ± 0.5	39.6 ± 0.5	-1.85	39.3 ± 0.5	39.5 ± 0.5	+0.82
MCV (um^3^)	85.6 ± 1.1	84.4 ± 1.1	-1.36	87.2 ± 0.9	87.2 ± 1.0	-0.03
MCH (pg)	28.9 ± 0.4	29.0 ± 0.4	+0.44	29.3 ± 0.4	29.8 ± 0.4	+2.02
MCHC (g/dl)	33.7 ± 0.2	34.3 ± 0.2^†^	+1.81	33.6 ± 0.2	34.2 ± 0.2^†^	+2.15
RDW (%)	13.3 ± 0.2	14.0 ± 0.3	+5.76	13.3 ± 0.2	13.8 ± 0.2^†^	+4.60

Data represent least square mean ± SE. Differences were considered significant at *P *value < 0.05.

*indicates that baseline values of the soy group were significantly different from the baseline values of the control group

† signifies that final values were significantly different (*P *< 0.05) from their baseline values. No differences were observed between the soy and control groups when parameters where expressed as change (%) from baseline.

RBC = red blood cell; Hb = hemoglobin; Hct = hematocrit; MCV = mean corpuscular volume; MCH = mean corpuscular hemoglobin; MCHC = mean corpuscular hemoglobin concentration; RDW = red cell distribution width index.

**Table 5:** Effect of one year supplementation of soy or control foods on platelet parameters

Measures	Control (n = 27)			Soy (n = 35)		
	Baseline	Final	Change (%)	Baseline	Final	Change (%)
PLT (×10^3^/mm^3^)	278 ± 11	271 ± 11	-2.07	267 ± 10	274 ± 10	+2.82
MPV (um^3^)	9.17 ± 0.17	9.18 ± 0.17	+0.31	9.15 ± 0.15	9.16 ± 0.15	+0.44
PCT (%)	0.254 ± 0.009	0.247 ± 0.009	-1.58	0.242 ± 0.008	0.249 ± 0.008	+3.35
PDW (%)	17.3 ± 0.5	17.5 ± 0.5	+1.44	16.7 ± 0.4	16.7 ± 0.4	+0.96

Data represent least square mean ± SE.

No differences were observed between the soy and control groups when parameters where expressed as change (%) from baseline.

PLT = platelet count; MPV = mean platelet volume; PCT = plateletcrit; PDW = platelet distribution width.

## Discussion

Previous studies [20,21] indicate that estrogen deficiency results in alterations in hematological parameters. For example, Ben-Hur et al. [20] reported that postmenopausal women between the ages of 50 to 60 years had higher monocyte counts than women between 18 to 24 years of age. On the other hand, women, five years or more postmenopausal, have been reported to have lower lymphocyte numbers than both early postmenopausal (6 months to 2 years) and premenopausal women [21]. Hence, the present study was designed to examine whether daily consumption of 25 g soy protein, rich in isoflavones, by postmenopausal women for one year affected these parameters. We hypothesized that moderate intake of soy products containing isoflavones by postmenopausal women may positively modulate monocyte and lymphocyte counts. This hypothesis was based on the earlier findings by our group [18] and others [22] showing that isoflavones restored total and differential white blood cell counts to normal levels in ovarian hormone deficient rodent models. Whether such a positive effects of soy isoflavones on hematological parameters observed in rodent models [18,22] of ovarian hormone deficiency can be seen in postmenopausal women needed investigation.

Although we observed that soy normalized the differential white blood cell counts in our rat study [18], the findings of the present clinical study indicated that consumption of 25 g soy protein containing 60 mg isoflavones for one year had no effect on total and differential white blood cell counts in postmenopausal women. The differences in response to soy isoflavone supplementation between our human and rat studies may be due to the fact that the hematopoietic system in humans and rodents are affected differently in estrogen deficiency. Additionally, the ingested levels of isoflavone per kg body weight were considerably different between the two studies. The rats were fed a diet that delivered 25 mg isoflavone per kg body weight, whereas the postmenopausal women in our study received the equivalent of approximately 1.1 mg isoflavones/kg body weight.

In terms of monocytes, increased number of monocytes [20] has been observed when a woman reaches the age of menopause. Moreover, monocytes from postmenopausal women appear to be more susceptible to inflammation than premenopausal women as shown by higher levels of tumor necrosis factor (TNF)-α [23]. In the present study, although monocyte numbers were not altered by soy treatment, the percentage of monocytes tended (*P *< 0.087) to decrease when compared with their corresponding baseline values. However, this was not the case in the group that received the control regimen. This effect of soy isoflavones on monocytes may be similar to that of HRT which has been shown to attenuate the elevation of monocyte induced by estrogen deficiency [20]. Estrogen has been shown to reduce the production of inflammatory cytokines such as interleukin (IL)-1β, IL-6 and TNF-α in postmenopausal women [24]. Therefore, it is reasonable to speculate that similar to estrogen, soy isoflavones, which are considered natural selective estrogen receptor modulators (SERMs), may also reduce the synthesis of inflammatory molecules in ovarian hormone deficiency.

As for the effects of isoflavones on lymphocyte counts, reports [16,17] point to an undesirable lowering of lymphocytes after long-term isoflavone use. For instance, Alexandersen et al. [16] had reported that a number of women developed lymphocytopenia after daily intake of 600 mg ipriflavone for three years. In the present study, we did not observe changes in lymphocyte counts in postmenopausal women who consumed soy products delivering 60 mg isoflavones daily for one year. However, women in the present study received one tenth of the dose of isoflavones consumed by participants in the study by Alexandersen and colleagues [16]. We chose the lower dose because of its appropriateness for examining the effects of soy on bone [6,7], which was the focus of the current study. By comparison, the high doses of ipriflavone that raised concerns related to lymphocytopenia may be considered a pharmacological dose, which is not likely to be achieved with dietary soy supplementation.

Red cell distribution width index (RDW) and MCHC were elevated in soy group and control group had higher levels of only MCHC. The increase in MCHC, the ratio of hemoglobin to hematocrit, may be due to a numerical increase in hemoglobin concentration in the soy group and a numerical lower hematocrit value in the control group. Elevated RDW is associated with increased immature RBC or reticulocyte numbers [24]. Reticulocyte counts increase in pathological conditions such as anemia [24]. However, this should not be the case in the present study because values for both hemoglobin and MCHC were within normal ranges in the soy group. The observed increases in the mean basophil counts and percent in this study were not expected. The absolute basophil counts in both groups slightly exceeded the reference range of 0 to 0.15 (×10^3^/mm^3^) for healthy postmenopausal women [25]. However, there were no differences in percentage changes of basophil between soy and control groups and the women did not report any allergic reactions therefore, it can be concluded that the change in basophil counts observed with soy protein was not a consequence of an allergic reaction. Moreover, the changes in basophile counts may be associated with the high coefficient of variation associated with automated hematology analyzer. Whether the findings of this one year study on hematological parameters can also be extrapolated to isolated soy isoflavones need further evaluation.

## Conclusion

In summary, our findings suggest that soy protein with 60 mg its isoflavones does not significantly alter hematological parameters in postmenopausal women. However, it is necessary to further explore the impact, if any, of the changes in monocytes, basophils and reticulocytes observed with protein supplementation in this study.

## Competing interests

The author(s) declare that they have no competing interests.
